# Applying machine learning to consumer wearable data for the early detection of complications after pediatric appendectomy

**DOI:** 10.1038/s41746-023-00890-z

**Published:** 2023-08-16

**Authors:** Hassan M. K. Ghomrawi, Megan K. O’Brien, Michela Carter, Rebecca Macaluso, Rushmin Khazanchi, Michael Fanton, Christopher DeBoer, Samuel C. Linton, Suhail Zeineddin, J. Benjamin Pitt, Megan Bouchard, Angie Figueroa, Soyang Kwon, Jane L. Holl, Arun Jayaraman, Fizan Abdullah

**Affiliations:** 1 Department of Surgery, Northwestern University Feinberg School of Medicine, Chicago, IL USA; 2 Department of Pediatrics, Northwestern University Feinberg School of Medicine, Chicago, IL USA; 3 Center for Health Services and Outcomes Research, Northwestern University Feinberg School of Medicine, Chicago, IL USA; 4 Center for Global Surgery, Northwestern University Feinberg School of Medicine, Chicago, IL USA; 5 Department of Medicine (Rheumatology), Northwestern University Feinberg School of Medicine, Chicago, IL USA; 6 Shirley Ryan AbilityLab, Chicago, IL USA; 7 Division of Pediatric Surgery, Ann and Robert H. Lurie Children’s Hospital of Chicago, Chicago, IL USA; 8 Northwestern University Feinberg School of Medicine, Chicago, IL USA; 9 Department of Pediatrics, Ann and Robert H. Lurie Children’s Hospital of Chicago, Chicago, IL USA; 10 Department of Neurology and Center for Healthcare Delivery Science and Innovation, Biological Sciences Division, University of Chicago, Chicago, IL USA; 11 Department of Physical Medicine and Rehabilitation, Northwestern University Feinberg School of Medicine, Chicago, IL USA; 12 Department of Medical Social Sciences, Northwestern University Feinberg School of Medicine, Chicago, IL USA; 13 Department of Physical Therapy and Human Movement Sciences, Northwestern University Feinberg School of Medicine, Chicago, IL USA; 14 Division of Pediatric Surgery, Ann and Robert H. Lurie Children’s Hospital of Chicago, 225 East Chicago Avenue, Box 63, Chicago, IL 60611 USA

**Keywords:** Paediatric research, Outcomes research, Predictive markers, Diagnosis, Signs and symptoms

## Abstract

When children are discharged from the hospital after surgery, their caregivers often rely on *subjective* assessments (e.g., appetite, fatigue) to monitor postoperative recovery as objective assessment tools are scarce at home. Such imprecise and one-dimensional evaluations can result in unwarranted emergency department visits or delayed care. To address this gap in postoperative monitoring, we evaluated the ability of a consumer-grade wearable device, Fitbit, which records multimodal data about daily physical activity, heart rate, and sleep, in detecting abnormal recovery early in children recovering after appendectomy. One hundred and sixty-two children, ages 3–17 years old, who underwent an appendectomy (86 complicated and 76 simple cases of appendicitis) wore a Fitbit device on their wrist for 21 days postoperatively. Abnormal recovery events (i.e., abnormal symptoms or confirmed postoperative complications) that arose during this period were gathered from medical records and patient reports. Fitbit-derived measures, as well as demographic and clinical characteristics, were used to train machine learning models to retrospectively detect abnormal recovery in the two days leading up to the event for patients with complicated and simple appendicitis. A balanced random forest classifier accurately detected 83% of these abnormal recovery days in complicated appendicitis and 70% of abnormal recovery days in simple appendicitis prior to the true report of a symptom/complication. These results support the development of machine learning algorithms to predict onset of abnormal symptoms and complications in children undergoing surgery, and the use of consumer wearables as monitoring tools for early detection of postoperative events.

## Introduction

More than 3.9 million children undergo surgery each year in the United States^1^. With shortened lengths of stay for inpatient procedures and an increasing number of surgeries being performed as same-day surgeries, more children are discharged home shortly after surgery^2,3^. Parents and other caregivers, whom we refer to collectively as “caregivers”, inherently assume a postoperative monitoring role for their children, but with few tools that provide objective data about the child’s recovery. With children being less communicative and less accurate historians of their illness than adults, caregivers must often rely on subjective assessments, such as perceived well-being, appetite, or fatigue, as indicators of abnormal recovery and decide whether or not to seek care^4–6^. This model of post-discharge care has resulted in both unwarranted healthcare use^7–9^ and delays in seeking care leading to serious complications^10–15^. For example, studies have shown that 30–50% of emergency department visits that occur after pediatric appendectomy, the most common inpatient pediatric procedure, are potentially avoidable^7,8^.

Remote-monitoring tools, which collect information from patients in the comfort of their own homes and provide near real-time, objective data to clinicians, have been shown to alleviate caregivers’ burden and improve patient outcomes^16^. However, current remote-monitoring systems are expensive and rarely applied to surgical patients^16^. With recent advances in technology, data previously limited to expensive remote-monitoring tools are now available from widely accessible and affordable consumer-grade wearable devices, such as the Fitbit. These devices generate continuous, valid, and objective measures of heart rate (HR), physical activity (PA), and sleep^17^. “Less than expected” PA and sleep disturbances are often important indicators of altered recovery^18–20^. However, at present, PA and sleep have only been subjectively assessed, even by clinicians^21^. In addition, consumer-grade wearable devices transmit data in near real-time^22–26^, thus making them potentially affordable and scalable alternative remote-monitoring tools^27^.

To date, the use of data from a consumer-grade wearable device to characterize postoperative recovery in children remains largely unexplored. The large volume of data generated by these devices’ multiple sensors have been difficult to process and associate with clinically meaningful events^17^. Advances in machine learning (ML) methods are accelerating data analysis and interpretability^28^. Consumer-grade wearables and ML have already shown promise to improve clinical detection in many other domains, with models developed to predict cardiovascular diseases^29^ (including the detection of arrhythmias^30–33^, heart failure with reduced ejection fraction^34^, and disability following stroke^35^), exertional heat illness^36^, psychiatric disorders^37,38^, and infection^39–49^. Arguably, one of the greatest contributors to propagating consumer-grade wearable-based digital biomarkers as part of multimodal risk prediction models was the COVID-19 pandemic. Multiple ML models have been designed with features derived from consumer wearable devices, often supplied by the patient, arising from the need to remotely monitor quarantined individuals in a resource-efficient manner^40,41,44–49^. Even with the growing prevalence of consumer-grade wearables and ML in clinical detection, most of these models have been developed using data recorded from adults with little work being done in children^50–58^.

In this study, we evaluated consumer-grade wearable devices, the Fitbit Inspire HR and Inspire 2, as postoperative remote-monitoring tools for children after appendectomy. We applied ML methods to Fitbit data to understand the underlying patterns in PA, HR, and sleep associated with abnormal symptoms and complications. We hypothesized that patients’ PA, HR, and sleep patterns, measured using a consumer-grade wearable and evaluated using ML, can detect postoperative recovery days with abnormal symptoms and/or complications early, i.e., before they occur/are reported.

## Results

### Patient characteristics

**Fig. 1:**
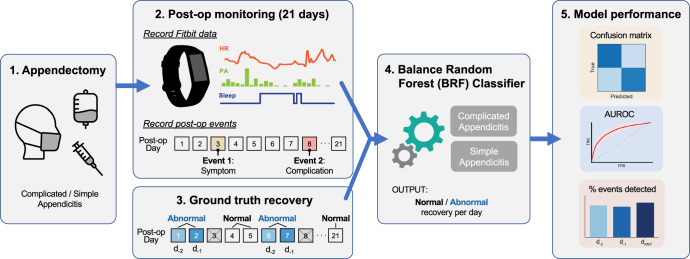
Study overview. Pediatric patients were given a Fitbit for 21 days following appendectomy for complicated or simple appendicitis to record physical activity (PA), heart rate (HR), and sleep data. Postoperative events (i.e., abnormal symptoms or confirmed complications) were identified from medical records and patient reports and used to label the 2 days prior to the event as “abnormal” and all other days as “normal” to indicate ground truth recovery. The days of reported events were excluded from the ground truth. Balanced random forest classifiers were trained to predict normal/abnormal recovery, with separate models for patients with complicated and simple appendicitis. Model performance was evaluated using confusion matrices, Area Under the Receiver Operating Characteristic curve (AUROC), as well as the percentage of events that were detected two days prior to the event (d_−2_), 1 day prior to the event (d_−1_), or on either day (d_ANY_; total number of events detected 1–2 days prior to the event).

This study took place at Ann and Robert H. Lurie Children’s Hospital of Chicago. Between March 2019—May 2019 and February 2020–June 2022, 162 children, ages 3–17 years old, undergoing appendectomy for either complicated or simple appendicitis were recruited after surgery and enrolled after written informed consent was obtained. All cases in the study were performed laparoscopically. Non-ambulatory children and children with preexisting mobility limitations or postoperative activity limitations, children with comorbidities that could alter the postoperative course, and children with COVID-19 were excluded. Patients were enrolled in the study and monitored with a Fitbit wearable following surgery. Patients were a mean age of 10.4 years (standard deviation [SD] 3.6 years) and 47.9% were female. Patients were 25.3% non-Hispanic White, 57.4% Hispanic/Latinx, 8.6% African American, and 4.3% of other races. The average length of stay was 2.6 days (SD 2.5 days). One patient was excluded from the analysis due to only having one hour of Fitbit data. Of the remaining 161 patients, 85 (53%) were treated for complicated appendicitis and 76 (47%) for simple appendicitis. Characteristics of patients included in the analysis are described in Table 1. During the monitoring period, there were 41 postoperative events (abnormal symptoms or confirmed complications) among patients with complicated appendicitis, and there were 10 postoperative events among patients with simple appendicitis. A total of 74 postoperative days (4.7%) were labeled as “abnormal” recovery days for the complicated appendicitis group and 20 postoperative days (1.3%) were labeled as “abnormal” recovery days for the simple appendicitis group, defined as the 1–2 days prior to the reported postoperative event. These data were used to develop and train a ML model to detect abnormal recovery using a combination of Fitbit metrics, patient demographics, and clinical characteristics (Fig. 1). Separate models were developed for patients with complicated and simple appendicitis.

**Table 1:** Demographics for a cohort of children <18 years old who underwent appendectomy stratified by type of appendicitis (complicated or simple) from 2019–2022 at a tertiary children’s hospital.

	Total (*n* = 161)	Patients with complicated appendicitis (*n* = 85)	Patients with simple appendicitis (*n* = 76)
Length of stay in days, mean (SD)	2.6 (2.5)	4.0 (2.6)	0.9 (0.7)
Age in years, mean (SD)	10.4 (3.6)	10.4 (3.7)	10.4 (3.5)
Sex			
Female, *n* (%)	78 (48.4)	44 (51.8)	34 (44.7)
Male, *n* (%)	83 (51.6)	41 (48.2)	42 (55.2)
Race/ethnicity			
Non-hispanic, white, *n* (%)	41 (25.5)	19 (22.4)	22 (28.9)
Hispanic/Latinx, *n* (%)	93 (57.8)	53 (62.4)	39 (51.3)
African American, *n* (%)	14 (8.7)	7 (8.2)	7 (9.2)
Other, *n* (%)	7 (4.3)	4 (4.7)	3 (3.9)

*SD* standard deviation.

### ML model predicts abnormal recovery days with high sensitivity

**Fig. 2:**
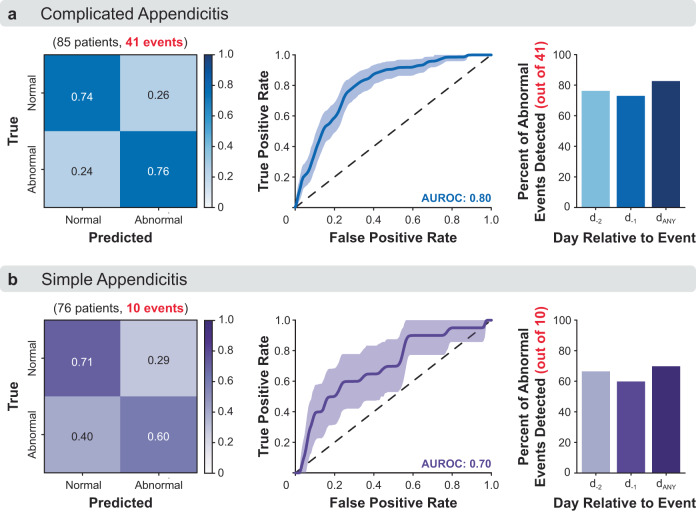
Model performance for early detection of abnormal recovery. Confusion matrix, receiver operating characteristic, and percent of postoperative events (either confirmed complications or abnormal symptoms) detected for appendectomy patients with **a** complicated appendicitis and **b** simple appendicitis.

A balanced random forest (BRF) algorithm was selected for having higher sensitivity (better recall) than other candidate models to detect “abnormal” recovery days in both the complicated and simple appendicitis groups (Supplementary Table 1). Confusion matrices, Receiver Operating Characteristic (ROC) curves, and percentage of detected events of the BRF models are summarized in Fig. 2. Precision-Recall (PR) curves^59^ are shown in Supplementary Figure 1. For patients with complicated appendicitis (Fig. 2a), 74% and 76% of “normal” and “abnormal” days were correctly identified, respectively. The classifier demonstrated very good predictive power with an AUROC of 0.80 (90% confidence interval (CI) [0.76–0.83]). Of the 41 postoperative events for this group, 83% were detected within the two days prior to their reported occurrence, with 76% detected 2 days prior to their reported occurrence (excluding events occurring within the first 2 days after surgery) and 73% detected 1 day prior to their reported occurrence (excluding events occurring one day after surgery). There were 387 false positives in the complicated appendicitis group, and the area under the PR curve (AUPRC) was 0.36 (90% CI [0.11–0.82]; Supplementary Fig. 1a). This outperforms a classifier with random performance, which would have an approximate AUPRC of 0.05 for the complicated appendicitis dataset based on the proportion of positive samples for this group (74 abnormal days out of 1581 total days available for model training).

For patients with simple appendicitis (Fig. 2b), 71% and 60% of “normal” and “abnormal” days were correctly identified, respectively. The classifier demonstrated good predictive power with an AUROC of 0.70 (90% CI [0.60–0.80]). Of the 10 postoperative events for this group, 70% were detected within the two days prior to their reported occurrence, with 67% detected two days prior to their reported occurrence (excluding events occurring within the first two days after surgery) and 60% detected one day prior to their reported occurrence (excluding events occurring one day after surgery). There were 438 false positives in the simple appendicitis group, and the AUPRC was 0.03 (90% CI [0.02–0.05]; Supplementary Fig. 1b). This only slightly outperforms a classifier with random performance, which would have an approximate AUPRC of 0.01 for the simple appendicitis dataset based on the proportion of positive samples for this group (20 abnormal days out of 1536 total days available for model training).

Of the 51 total postoperative events, 10 (19.6%) were missed by these models. For complicated appendicitis, there were 7 missed events, including 5 Grade I and 2 Grade III (intraabdominal abscess requiring drain placement) according to the Clavien-Dindo scale. For simple appendicitis, there were 3 missed events, including 2 Grade I and 1 Grade III (intraabdominal abscess requiring surgical washout and drain placement). All other events, ranging from Grades I–III, were successfully detected by the model (Supplementary Table 2). The distribution of detected and missed events as a function of postoperative day is shown in Supplementary Figure 2.

**Fig. 3:**
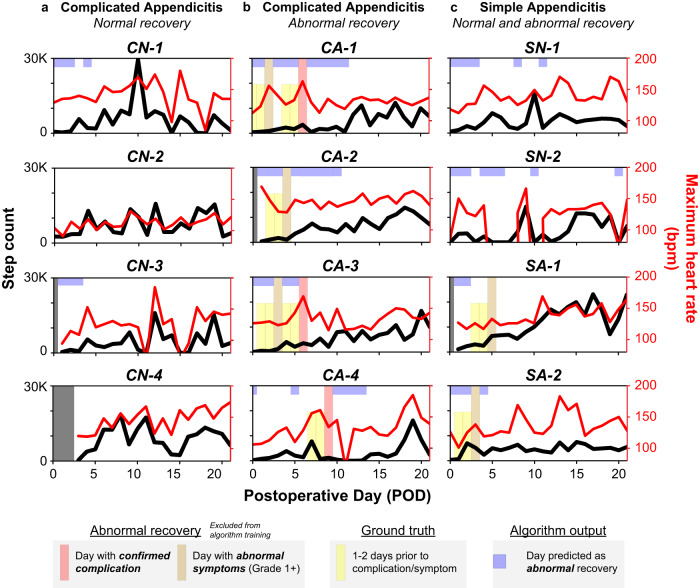
Patient case studies. Example Fitbit data, ground truth for early detection, and ML predictions for representative patients during the 21-day monitoring period. Daily step count (black line) and maximum heart rate (red line) are obtained from the Fitbit. Abnormal recovery days predicted by the ML classifier are shown as blue boxes above the ground truth label for each day (yellow bars for the 1-2 days prior to the reported abnormal symptom/complication, or no bar for normal recovery). **a** Four patients with complicated appendicitis who had a normal recovery. False positives do occasionally occur, typically in the immediate perioperative period or during periods of low activity as seen in patients CN-1 and CN-3. **b** Four patients with complicated appendicitis who experienced abnormal postoperative events, three of which were intraabdominal abscesses managed with intravenous antibiotics (patients CA-1 and CA-3) or drainage procedures (patient CA-4). **c** Four patients with simple appendicitis, two patients (SN-1 and SN-2) with normal recovery, and two patients (SA-1 and SA-2) with abnormal postoperative events. Abnormal symptoms generally occurred within the first five postoperative days in this group, with only 20% occurring later than POD 5.

Representative case studies, showing Fitbit data for individual patients during the postoperative monitoring period compared to their actual recovery and model predictions, are shown in Fig. 3. In the complicated appendicitis group, the model identified patients who experienced normal recovery (Fig. 3a) with approximately the same accuracy as those who had an abnormal recovery (Fig. 3b). Model predictions of “abnormal recovery” on days when the ground truth was “normal recovery” generally occurred in the days immediately after an abnormal event, as shown in patients CA-1, CA-2, and CA-4, and in periods of low activity level, as shown in patients CN-1, CN-3, and CA-4. For simple appendicitis (Fig. 3c), the model tended to favor temporal clinical features and generally predicted “abnormal recovery” during the first few days after surgery regardless of whether there was a true deviation from normal recovery.

**Fig. 4:**
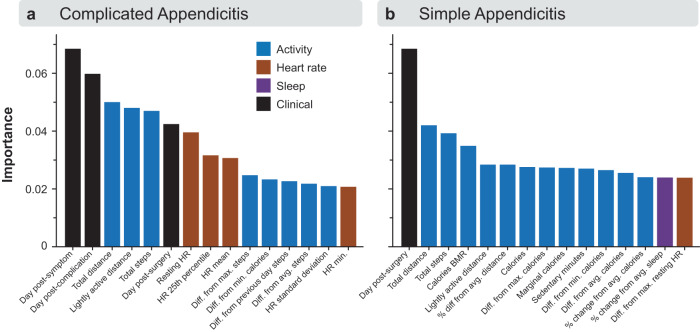
Model feature importance. The top 15 features, averaged across all cross-validation folds, are shown for **a** complicated and **b** simple appendicitis groups. The complicated appendicitis model used a combination of clinical characteristics, activity, and heart rate data. The simple appendicitis model relied primarily on clinical characteristics and activity data.

The average Gini feature importance showed that, in the complicated appendicitis model (Fig. 4a), the two most important features were the number of days after a previous symptom/complication, followed by total distance and lightly active distance estimated from the Fitbit. The simple appendicitis model (Fig. 4b) relied more heavily on the number of days post-surgery, followed by total distance and total steps estimated from the Fitbit.

### Sensitivity analysis 1: removing fitbit data features

**Fig. 5:**
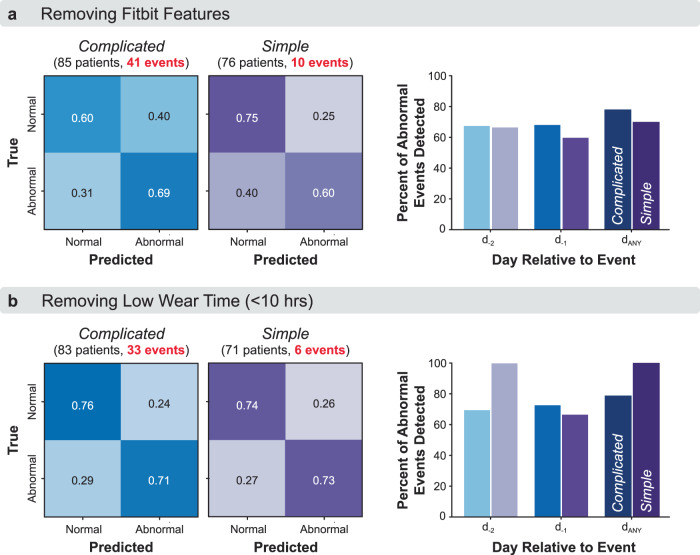
Sensitivity analyses of Fitbit features and device wear time. Model performance for patients undergoing appendectomy for complicated and simple appendicitis when the classifier was trained by **a** removing features computed from Fitbit data and using only clinical characteristics and demographic features, or **b** removing days with low Fitbit wear time (<10 h).

Removing Fitbit features from the classifier decreased performance for complicated appendicitis (correctly detecting 60% of “normal” and 69% of “abnormal” days for this group) but did not substantially affect performance for simple appendicitis. Without Fitbit data, 78% and 70% of postoperative events were detected up to two days prior to their reported occurrence for complicated and simple appendicitis, respectively (Fig. 5a).

### Sensitivity analysis 2: removing days with low wear time

Patients varied in their Fitbit wear time. Average daily wear time was 12.8 ± 6.0 h and 12.3 ± 6.3 h for the complicated and simple appendicitis groups, respectively. When restricting the analysis to days when the Fitbit device was worn at least 10 hours/day during typical waking hours (6:00 am–12:00 am), 24% and 26% of the monitored days were excluded for the complicated and simple appendicitis models, respectively. Removing days with low wear time did not substantially affect model performance for complicated appendicitis, but it did improve performance for simple appendicitis (correctly detecting 74% of “normal” and 73% of “abnormal” days for this group). Without days with low wear time, 79% and 100% of postoperative events were detected up to two days prior to their reported occurrence for complicated and simple appendicitis, respectively (Fig. 5b).

### Sensitivity analysis 3: removing different Fitbit device types

Most participants wore the Fitbit Inspire HR during the monitoring period, including 69 patients with complicated appendicitis (who had 31 postoperative events) and 43 patients with simple appendicitis (who had only 3 postoperative events). Using data from this device alone (removing the Inspire 2) did not substantially change model performance for complicated appendicitis but decreased performance for simple appendicitis (correctly predicting 62% of “normal” and 20% of “abnormal” days for this group). With only the Inspire HR, 77% and 33% of postoperative events were detected up to two days prior to their reported occurrence for complicated and simple appendicitis, respectively (Supplementary Figure 3a).

Of remaining participants who wore the Fitbit Inspire 2, 16 had complicated appendicitis (with 10 postoperative events), and 33 had simple appendicitis (with 7 postoperative events). Using data from this device alone (removing the Inspire HR) slightly decreased model performance for complicated appendicitis (correctly predicting 60% of “normal” and 67% of “abnormal” days) but increased for the simple appendicitis group (correctly predicting 75% of “normal” days and 67% of “abnormal” days). With only the Inspire 2, 70% and 71% of postoperative events were detected up to two days prior to their reported occurrence for complicated and simple appendicitis, respectively (Supplementary Figure 3b).

### Sensitivity analysis 4: combined model for complicated and simple appendicitis

In an alternative model combining data from the complicated and simple appendicitis groups, 74% and 70% of “normal” and “abnormal” days were correctly identified, respectively. The classifier demonstrated good predictive power with an AUROC of 0.79. Of the 51 postoperative events for this group, 78% were detected within the two days prior to their reported occurrence, with 70% detected two days prior to their reported occurrence, and 69% detected one day prior to their reported occurrence (Supplementary Figure 4). Compared to the separate models for complicated and simple appendicitis, one additional postoperative event was missed in this combined model (11 missed events total; including an additional Grade I event in a patient with simple appendicitis).

## Discussion

Here we report the use of the Fitbit wearable device to monitor children after appendectomy, the most common inpatient pediatric surgical procedure in the U.S., and applied ML methods to retrospectively detect abnormal recovery prior to a reported postoperative event. Our models detect more than 70% of postoperative events (abnormal symptoms and confirmed complications) in the two days prior to their reported occurrence in patients undergoing appendectomy for both complicated and simple appendicitis. Fitbit-based models are more effective for patients with complicated appendicitis, detecting 83% of all postoperative events in this cohort. These findings support the use of commercial wearables as potential remote-monitoring tools for children recovering from surgery, such as from a laparoscopic appendectomy for complicated appendicitis.

This study sought to generate clinically relevant ML models for the detection of abnormal recovery and complications after appendectomy in children. Although there have been efforts in adult patients after surgery, little has been done in children^60,61^. The ability of a model to detect up to 83% of abnormal events up to 2 days before they are reported by the caregiver has the potential to dramatically improve patient outcomes. For example, infection is one of the most frequent and expensive complications after appendectomy. Infection leading to sepsis is a major cause of readmissions and is associated with significantly higher healthcare costs and worse patient outcomes^62,63^. However, early detection of infection has been associated with better outcomes; therefore, Fitbit data could lead to better patient outcomes and lower healthcare costs^64,65^.

The implementation of a Fitbit-based remote-monitoring system has the potential to dramatically improve post-discharge healthcare in the U.S. The current care model provides patients and caregivers with instructions for identifying concerning symptoms and signs of infection after discharge. This inherently assumes that patients and their caregivers have appropriate clinical knowledge, are continuously monitoring, and are free of biases as to when to contact their healthcare team. Under this care model, contact with the healthcare system is always “patient-initiated”. This may contribute to existing disparities in outcomes. An objective, continuous monitoring system based on daily measures of health and activity from a wearable device would allow clinical teams to not only gain unprecedented insight into patient recovery in near real-time, but would also enable them to reach out to patients in a timely manner. This expands the current post-discharge care model so it includes “health system-initiated” contact with the patient. This may facilitate more timely detection and treatment of abnormal symptoms and postsurgical complications and may reduce existing disparities.

It is important to note that while our Fitbit-based ML model is promising, additional work is still needed. The models presented in this paper have a non-trivial rate of false positives, especially during the first few days after surgery. Lower values of AUPRC reflect the relatively high number of false positives, but the model also has high sensitivity to detect abnormal postoperative events. It is also possible that what we consider to be false positives are actually true instances of abnormal recovery that were not reported by the caregiver during the phone screenings. This is supported by multiple cases where the model predicts more days as “abnormal” coinciding with the patient reporting more severe symptoms during the monitoring period. In a real-world clinical application, detecting true positives has a much higher benefit to care than minimizing false alarms. This is especially true given an alert could be a short phone call or text message to the patient’s caregiver to check on their child. However, reducing these false alarms is important in the future because a high percentage of these alarms may quickly create alert fatigue and numb the response of the clinical team over time. Given that most missed events were abnormal symptoms and not NSQIP-verified complications, we believe that the current false positive rate represents an acceptable starting point and will likely decrease with more training data and continued model refinement. It is also important to recognize that alerts generated by a wearable-based early detection model may be perceived as additional work by clinicians. Redesigning the clinical workflow is necessary to incorporate these data in a meaningful way that does not impede the current workflow of clinical teams.

Our findings suggest that Fitbit data may not have the same value for all surgical procedures. The complicated appendicitis model weighs Fitbit data more highly than the simple appendicitis model. Indeed, models trained with or without Fitbit data perform similarly for patients with simple appendicitis, demonstrating that the classifier relies more heavily on other clinical indicators (i.e., postoperative day) than Fitbit data for these patients. We should note, however, that AUPRC values indicate that the simple appendicitis model does not perform much better than a random classifier. While it is possible that the low number of postoperative events available for model training may limit performance in this group (i.e., more data may improve algorithm performance), it is also possible that Fitbit data simply may not be as valuable to predict abnormal events in this patient cohort. Also, while it is encouraging that a model combining complicated and simple appendicitis performs similarly well to detect postoperative events across these two patient cohorts (Supplementary Figure 4), recognizing the populations that would benefit the most from a Fitbit-based monitoring system will be key to successful implementation in a real-world clinical setting.

Our study has several limitations which should be considered. Recruitment was limited to working days of the week, and there was a period of pause in recruitment. However, given the emergent nature of appendectomy, we anticipate that appendicitis patients who had their operation earlier in the study or those who had their operation on a weekend would have demographic and complication profiles similar to the study population. Due to the acute presentation of appendicitis and the emergent nature of laparoscopic appendectomy, we are unable to capture recovery using Fitbit data relative to pre-surgical baseline values. Previously, we investigated different strategies to overcome this limitation, such as acquiring Fitbit data from a community-recruited, healthy control population matched on sex, age, and weight to obtain a representative baseline sample for children who undergo emergent surgery; however, most activity metrics were different^66^. Furthermore, as we have observed that children who undergo laparoscopic appendectomy for complicated appendicitis can sometimes take more than 21 days to return to a statistically-derived baseline, a longer monitoring period would be needed to obtain a relative baseline after operation^67^. In the present study, we include a subset of Fitbit features based on changes in metrics from previous days. This approach may offer an alternative to account for a patient’s relative recovery over time if baseline data is not available. This a single-center study, and, as such, the patient population and discharge protocols, which may affect PA of patients, were ubiquitous. The ML models developed in this study population may not be generalizable to all patients undergoing appendectomy in the U.S., and further validation is needed in larger and more diverse patient populations across multiple institutions. Although developing separate models for complicated and simple appendicitis decreases the volume of data available for training and testing each model, we believe this is the most clinically appropriate approach given the expected differences in postoperative recovery for the two cohorts^67^. Future work will increase the sample size for model training and examine generalizability of the models using larger, held-out test sets. Incorporating Fitbit data into remote health monitoring has benefits and drawbacks. While the device is more readily available and recognized in the U.S. consumer market and is comfortable and safe for children (even young children) to wear, it does not allow access to its raw data. Despite that, the Fitbit is practical and fairly accurate compared with clinical-grade devices^68–77^. However, there are challenges. During the prolonged recruitment period of this study, the Fitbit Inspire HR was replaced in the consumer market by the Fitbit Inspire 2 necessitating a change in device. Our sensitivity analysis reveals the best-performing model corresponded to the device that had a majority (≥70%) of postoperative events for each group. While we cannot confirm Fitbit’s proprietary algorithms were consistent between device generations, these results suggest that data availability, rather than device type, is the primary determinant of model performance. Even so, our results may not generalize to other consumer wearables which use different hardware and software to obtain PA, HR, and sleep measures. Future work will focus on refining and validating a ML approach across different types of wearables, thereby developing models that are agnostic to inter-device variability. Furthermore, near real-time Fitbit monitoring requires access to a cell phone with a data plan for online synchronization with a clinical database. Though all caregivers approached in the study had a cell phone with a data plan, this requirement may be a barrier to participation for some patients and caregivers. Future work will investigate the accessibility and scalability of a Fitbit-based approach in other healthcare centers. The models developed in this study do not distinguish between the severity of postoperative events when examining onset of symptoms. Since this study is a first step to developing a meaningful screening tool for the post-discharge setting, this was an intentional decision to maintain focus on binary (normal/abnormal) classification. In this context, the benefits of a highly sensitive model that identifies all potential complications outweigh the detriments of an error-prone severity classification system as a matter of patient safety. Clavien-Dindo Grade IV complications are exceedingly rare after appendectomy (<1%), and we do not capture this type of complication in the current dataset. However, one patient with complicated appendicitis was found to be in septic shock at the initial presentation; therefore, she required a stay in the intensive care unit perioperatively. Although her illness was an extreme presentation of complicated appendicitis rather than a post-operative complication, her physiologic state was similar to a patient experiencing a Grade IV complication. This patient is flagged by the model for the first 7 postoperative days, and since the model is designed with high sensitivity to capture minor events, we expect it would function similarly should a Grade IV complication occur. Lastly, recall bias and frequent reporting of symptoms during the phone surveys may have an unintended consequence of parents seeking more and/or earlier medical care, thus decreasing the impact of true complications. As the dataset continues to grow, more data will be available across different symptoms and complications which could then be used to distinguish between postoperative event severities.

This study demonstrates practical use of the Fitbit, a widely available consumer-grade wearable, to detect abnormal recovery symptoms and complications in pediatric appendectomy patients up to two days before they occur. Further testing in larger cohorts of patients is warranted to refine the ML models as an important next step in the evaluation of this technology for postoperative remote monitoring.

## Methods

### Study setting and study population

After receiving approval from the Ann and Robert H. Lurie Children’s Hospital of Chicago (LCH) institutional review board (IRB #2018-1836), children, ages 3–17 years old, who had just undergone appendectomy for complicated or simple appendicitis at LCH and their caregivers were recruited for the study between March 2019–May 2019 and February 2020–June 2022. The interruption in recruitment was due to the unexpected loss of the study coordinator. The lower age limit of three years old was selected because prior studies demonstrated poor compliance with wearing the Fitbit device and patient dissatisfaction at younger ages^78^. Patients and their caregivers were recruited shortly after surgery, and written informed consent using IRB-approved forms was obtained from a parent or legal guardian for all children <18 years old. In addition, oral assent was obtained from patients 7–11 years old and written assent from patients 12 years and older.

### Data sources and collection

Eligible patients and their caregivers were identified by daily review of the electronic health records (EHR), recruited, and enrolled by the study coordinator. The Fitbit Inspire HR and Fitbit Inspire 2 were chosen for their reliable use in children^68,69,75,78^, and because of our own experience showing high compliance in children^67^. All patients were approached immediately after being sent to their hospital room during daytime working hours (7 am–5 pm) or the next morning for those who underwent surgery at night. Recruitment occurred in the patient’s room after return from the recovery unit and emergence from general anesthesia to maximize patient participation. Patients with simple appendicitis who had an evening operation and were discharged home that evening were not recruited. Patients who had their surgery and discharged during the weekend were also not recruited. After agreeing to participate in the study, the study coordinator placed the Fitbit on the patient’s wrist, demonstrated appropriate use, and assisted with registration of the child’s Fitbit on their caregiver’s smartphone with a Fitbit account. This account was then linked to Fitabase, a cloud-based platform that receives Fitbit data in near real-time from the caregiver’s smartphone. Patients were instructed to wear the device continually on either wrist for 21 postoperative days, whether hospitalized or discharged home. Compliance with wearing the Fitbit was monitored by the study coordinator through Fitabase on a daily basis. If data were not synchronized during the preceding 18 h, the patient/caregiver was contacted. At the end of the follow-up period, Fitbit was given to participants as remuneration for their participation in the study.

Clinical information about the surgery (surgery type, surgery date, and hospital discharge) and demographics were gathered from the patient’s EHR. Information about any symptoms and complications that occurred after surgery were abstracted from the EHR during the index hospitalization and any subsequent ED visits, outpatient visits, calls to the hospital, readmissions, and from patients and/or caregivers via phone surveys conducted on POD 3, 7, 10, 14 and 21 for outpatients. A standardized symptoms checklist was utilized to inquire about these events. The details of this information are described in the next section.

### Categorization of surgery and clinical events

An appendicitis was categorized as simple if no presence of perforation, phlegmon, or abscess and complicated if perforation, phlegmon, or abscess was present at surgery. Simple and complicated appendicitis patients have significantly different postoperative recovery trajectories due to the greater disease severity in the case of complicated appendicitis, which more frequently requires additional inpatient treatments (e.g. antibiotics, intravenous fluids), prolonged return to normal bowel function, increased pain, and occasionally additional invasive procedures (percutaneous drainage of abscess), compared to simple appendicitis^79,80^.

Postoperative events were reviewed and categorized using the validated Clavien-Dindo classification system and the American College of Surgeons’ National Surgical Quality Improvement Program (ACS NSQIP) list of complications^81,82^. Events were categorized as “abnormal” if they were outside the expectations of normal recovery. To understand the breadth of severity of abnormal events, a Clavien-Dindo grade was assigned to each abnormal event (Table 2). By definition, all Grade ≥I events are outside of expectations for normal recovery. A “complication” was defined based on the NSQIP definition, which in this study included deep and superficial site infection, small bowel obstruction, unplanned return to the operating room, and *Clostridium difficile* infection requiring readmission. All other reported symptoms were considered as within the scope of “normal recovery” (i.e., events requiring no additional therapies or interventions beyond standard perioperative and discharge protocols). All symptoms, complications, and their corresponding categorizations were reviewed and confirmed by a senior pediatric surgeon (FA) and 4 surgery residents (CD, SCL, JBP, and MC).

**Table 2:** Clavien-Dindo classification grading with corresponding postoperative events available for model development and testing.

Clavien-Dindo grade description:	Examples	Frequency (*n*) in patients with complicated appendicitis	Frequency (*n*) in patients with simple appendicitis
Grade I: Any deviation from normal postoperative course without the need for surgical, endoscopic, and radiological interventions. Allowed therapeutic regimens include antiemetics, antipyretics, analgesics, diuretics, electrolytes, and physiotherapy.	Fever requiring antipyretic, incisional redness/drainage not requiring antibiotics, vomiting requiring antiemetic, oliguria requiring foley catheter, diarrhea requiring IV hydration	22	9
Grade II: Requiring pharmacological treatment with drugs other than such allowed for grade I complications	Ileus requiring nasogastric tube and total parental nutrition (TPN), surgical site infection requiring antibiotics*, intraabdominal abscess treated with IV antibiotics alone*, Clostridium dificile infection*	14	0
Grade III: Requiring surgical, endoscopic, or radiological intervention	Interventional radiology percutaneous drainage of intraabdominal abscess*, takeback to operating room for early adhesive small bowel obstruction*	5	1
Grade IV: Life-threatening complication requiring ICU management including single or multi-organ dysfunction.	No occurrences	0	0
Grade V: Death of the patient	No occurrences	0	0

*Also designated as an NSQIP complication.

### Ground truth labels

For every patient, each day of postoperative monitoring was labeled as either “abnormal” or “normal” recovery. A day was considered “abnormal” if it was within the 2 days leading up to a newly reported postoperative event (abnormal symptom or confirmed complication). This two-day detection period was chosen as a practical timeline for meaningful clinical intervention before the event would have been reported. The day of the postoperative event was excluded from model training and testing to prioritize early detection during the potential onset of the symptoms. For postoperative events that occurred on POD 3 or later, the two days prior to the postoperative event were labeled as “abnormal.” For events that occurred on POD 2, only the day prior to the event was labeled as abnormal, as there was data available two days prior to the event. For events that occurred on POD 1, there were no days prior to the event; therefore, no days leading up to the event were labeled as abnormal. All remaining days were labeled as “normal” recovery.

### Features extracted from the Fitbit data

Table 3 summarizes the 75 features extracted from the Fitbit data and uploaded into Fitabase. The minute-by-minute HR data were used to compute the maximum, minimum, average, and standard deviation of HR each day. Daily PA (e.g., steps, distance traveled, calories burned) and sleep data (e.g., time asleep, time in bed), computed using Fitbit’s proprietary algorithms, were also extracted^83–85^. Additional features were computed to capture temporal variations of the data, including changes from the previous day and changes from a 3-day rolling average, as outlined in Table 3. We also incorporated demographic and clinical characteristics, such as discharge status and the number of days since surgery or a previous symptom/complication.

**Table 3:** Features extracted and used in the machine learning model.

Category	Feature	Description (per day)
Activity	Total steps^Δ,δ,%,Μ,μ^	Steps taken
	Total distance^Δ,δ,%,Μ,μ^	Kilometers traveled
	Logged activities distance	Kilometers from logged activities
	Very active distance	Kilometers traveled during very active activities
	Moderately active distance	Kilometers traveled during moderate activity
	Light active distance	Kilometers traveled during light activity
	Sedentary active distance	Kilometers traveled during sedentary activity
	Very active minutes	Total minutes spent in very active activity
	Fairly active minutes	Total minutes spent in fairly active activity
	Lightly active minutes	Total minutes spent in light activity
	Sedentary minutes	Total minutes spent in sedentary activity
	Calories^Δ,δ,%,Μ,μ^	Total estimated energy expenditure
	Calories BMR	Total energy expenditure from basal metabolic rate
	Marginal calories	Total marginal estimated energy expenditure
Heart rate	Resting heart rate^Δ,δ,%,Μ,μ^	Average resting heart rate value
	Heart rate mean	Average heart rate value
	Heart rate standard dev.	Standard deviation of heart rate
	Heart rate minimum	Minimum heart rate
	Heart rate maximum	Maximum heart rate
Sleep	Total minutes asleep^Δ,δ,%,Μ,μ^	Total minutes asleep
	Total minutes in bed^Δ,δ,%,Μ,μ^	Total minutes in bed awake or asleep
	Total minutes restless^Δ,δ,%,Μ,μ^	Total time in bed awake
	Total sleep records	Number of sleep periods (>1 h)
Demographics	Age	Patient age at time of surgery
	Weight	Patient weight at time of surgery
	Height	Patient height at time of surgery
	Sex	Patient sex (male/female)
	Race/ethnicity	Patient race/ethnicity
Clinical characteristics	Days post-surgery	Days since surgery
	Days post-symptom	Days since last reported symptom
	Days post-complication	Days since last complication
	Past symptom	Boolean—has patient had a symptom
	Past complication	Boolean—has patient had a complication
	Number of past symptoms	Number of past symptoms
	Number of past complications	Number of past complications
	Discharged	Boolean—has patient been discharged

Δ – difference from the previous day; δ – difference from 3-day rolling average; % – percent change from rolling average; Μ – difference from maximum value of all previous days; μ – difference from minimum value of all previous days.

### ML models to classify normal or abnormal recovery days

Figure 1 summarizes the study pipeline for supervised ML using the Fitbit data features and recovery ground truth labels. Multiple supervised ML techniques were explored to determine the best-performing algorithm for this imbalanced learning problem, since complications were rare events compared to the number of total days monitored across patients. Adaptive boosting, eXtreme Gradient Boosting (XGBoost), random under-sampling (RUSBoost), balanced bagging, easy ensemble, and balanced random forest (BRF) classifiers were all tested. The BRF classifier outperformed other algorithms, with greater sensitivity (i.e., recall) to detect “abnormal” recovery days, and was selected for final model training and testing (Supplementary Table 1).

Given known differences in disease etiology and recovery trajectories^67^, we trained ML models separately for patients undergoing appendectomy for complicated and simple appendicitis. For each model, a BRF ensemble estimator was used to classify each labeled day as “normal” or “abnormal.” The BRF classifier fits an ensemble of decision trees on random subsamples of the dataset using bootstrap sampling, while also under-sampling the majority class (with replacement) on each bootstrapping iteration to balance the classes^86^. The random forest algorithm facilitates high-accuracy classification with a low number of hyperparameters, the ability to handle high-dimensional data, and robustness to outliers, while balanced under-sampling can improve performance for imbalanced datasets^87^ in this case, with fewer abnormal recovery days relative to normal recovery days. We used leave-one-subject-out cross-validation, wherein the model was iteratively trained using daily Fitbit features and ground truth labels from all patients but one and tested on the left-out patient.

### Evaluation of the model performance

AUROC and AUPRC were used to evaluate the performance of the model when predicting days labeled as “normal” or “abnormal” recovery. Additionally, we calculated the percent of postoperative events (reported occurrence of abnormal symptoms or confirmed complications) that were detected two days prior to the event (d_−2_), 1 day prior to the event (d_−1_), and on either of the 2 days prior to the event (d_ANY_). These metrics were averaged across all cross-validation folds. The average Gini impurity, a measure of an individual feature’s ability to correctly classify a day as “normal” or “abnormal,” was computed across all cross-validation folds. This enabled the features to be ranked by their relative importance to the model^88^.

Data reduction and imputation methods were applied during model estimation. To reduce the risk of overfitting, highly correlated features (Pearson’s correlation coefficient >0.95) were removed. For the initial models, days in which *any* Fitbit data were recorded were included in the analysis. This resulted in missing feature values on days with incomplete data (e.g., activity data recorded but no sleep data), as well as missing feature values that could not be computed due to an insufficient amount of wear time up to that point (e.g., computing changes in steps from a 3-day rolling average on days 1–3 post-surgery). These missing values were imputed using the patient’s mean value for that feature across the entire monitoring period.

Four sensitivity analyses were conducted to examine the impact of (1) Fitbit data availability, (2) device wear time, (3) device type, and (4) combined appendicitis groups on the model performance. First, the BRF classifiers were trained utilizing only the “clinical characteristics” and “demographics” features listed in Table 3 to evaluate the added value of including Fitbit data in the detection of abnormal recovery days. Second, the BRF classifiers were trained using only days in which the Fitbit device was worn at least 10 h per day during potential waking hours (6:00 am–12:00 am) to evaluate model performance when user compliance would be considered high to rule out the potential bias associated with data from a shorter wear time. The 10 h per day within the 6:00 am–12:00 am timeframe is the conventional threshold of wear-time required to estimate valid daily PA within a day. The device was considered as “not worn” if HR data (beats/minute) were zero or not recorded^67,89^. The classifier was not trained or tested on any excluded day in this analysis. However, all days with sufficient wear-time leading up to and following an excluded day were included with their original labels. Third, the BRF classifiers were trained utilizing data only from patients who used the Fitbit Inspire 2 (*n* = 49) or the Fitbit Inspire HR (*n* = 112). Finally, the classifiers were trained using combined data from patients in the complicated and simple appendicitis groups (*n* = 161).

### Reporting summary

Further information on research design is available in the Nature Research Reporting Summary linked to this article.

### Supplementary information

Supplemental Material

Reporting Summary

## Data Availability

The clinical data used in this study belongs to the Ann and Robert H. Lurie Children’s Hospital of Chicago, and restrictions apply to the availability of these data. Qualified researchers affiliated with the Ann and Robert H. Lurie Children’s Hospital of Chicago may apply for access to these data through the Ann and Robert H. Lurie Children’s Hospital of Chicago institutional review board.

## Code availability

The code for all the analysis conducted in this paper is available upon request to authors.
