# Supplementary material for: Applying machine learning to consumer wearable data for the early detection of complications after pediatric appendectomy
Source: NPJ Digit Med. 2023 Aug 16;6:148. doi: 10.1038/s41746-023-00890-z (PMC10432429; doi:10.1038/s41746-023-00890-z)
Supplement: Supplementary file 1 — Supplemental Material [file 41746_2023_890_MOESM1_ESM.pdf]

SUPPLEMENTARY MATERIAL

Applying Machine Learning to Consumer Wearable Data for the Early Detection of Complications After Pediatric Appendectomy

Authors: Ghomrawi HMK[δ], O'Brien MK[δ], Carter M, Macaluso R, Khazanchi R, Fanton M, DeBoer C, Linton SC, Zeineddin S, Pitt JB, Bouchard M, Figueroa A, Kwon S, Holl JL, Arun Jayaraman A*, Abdullah F*

[δ] Both authors contributed equally to first authorship.

*Both authors contributed equally to senior authorship.

Corresponding author:

Fizan Abdullah

Contact: fabdullah@luriechildrens.org

Supplementary Tables

Supplementary Table 1: Performance metrics for different machine learning classifiers. ADA, adaptive boosting; BB, balanced bagging; BRF, balanced random forest; EASY, easy ensemble; RUSB, random under-sampling (RUSBoost); XGB, eXtreme Gradient Boosting.

Complicated						
	ADA	XGB	RUSB	BB	EASY	BRF
Sensitivity	0.04	0.08	0.38	0.53	0.66	0.72
Specificity	0.98	0.98	0.82	0.81	0.72	0.75
Precision (weighted avg.)	0.92	0.92	0.93	0.93	0.94	0.94
Recall (weighted avg.)	0.94	0.94	0.82	0.81	0.72	0.74
F-1 score (weighted avg.)	0.92	0.93	0.86	0.86	0.80	0.82
Simple						
Sensitivity	0	0	0.25	0.45	0.55	0.60
Specificity	0.99	1.0	0.89	0.83	0.72	0.68
Precision (weighted avg.)	0.97	0.92	0.98	0.98	0.98	0.98
Recall (weighted avg.)	0.98	0.94	0.90	0.82	0.72	0.71
F-1 score (weighted avg.)	0.98	0.93	0.94	0.89	0.82	0.82

Supplementary Table 2: Number of detected and missed postoperative events by Clavien-Dindo grading.

Clavien-Dindo Grade	Total no. events	Complicated Appendicitis: Events detected (1-2 days prior)	Complicated Appendicitis: Events missed	Simple Appendicitis: Events detected (1-2 days prior)	Simple Appendicitis: Events missed
Grade I	31	17	5	7	2
Grade II	14	14	0	N/A	N/A
Grade III	6	3	2	0	1
Grade IV	0	N/A	N/A	N/A	N/A
Grade V	0	N/A	N/A	N/A	N/A
Total	51	34	7	7	3

Supplementary Figures

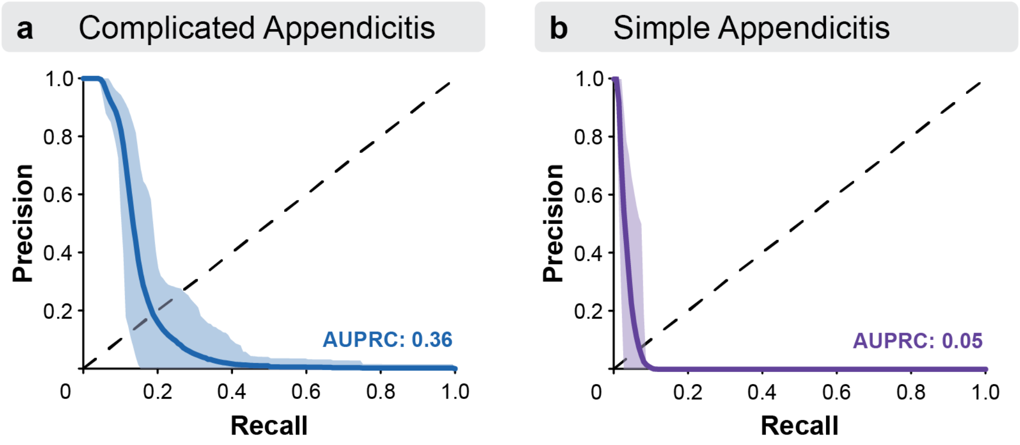

Supplementary Figure 1: Precision-Recall curves for early detection of abnormal recovery. Precision-recall curves for patients with (**a**) complicated appendicitis and (**b**) simple appendicitis. Baseline random classifier performance is approximated as 0.05 and 0.01 for these cohorts, respectively, based on the proportion of positive samples (abnormal recovery days) in the model training data.

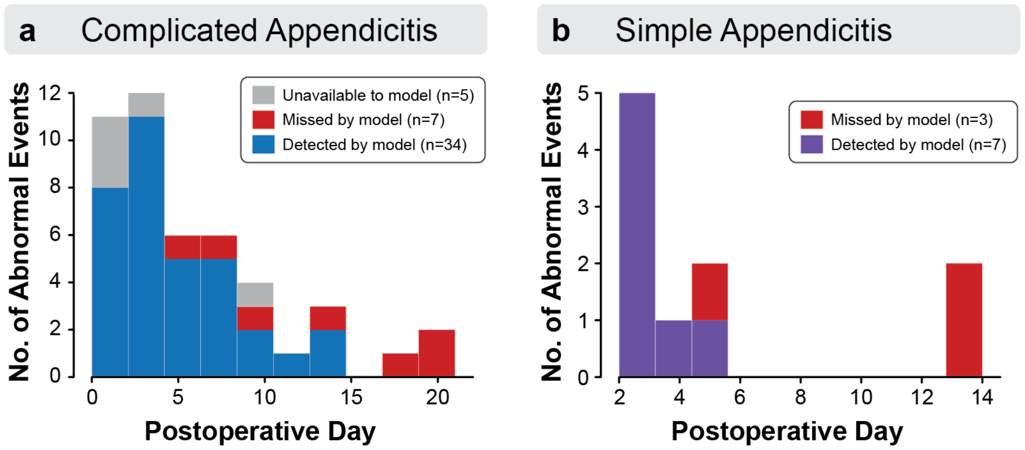

Supplementary Figure 2: Timing of postoperative events. The distribution of events (abnormal symptoms and confirmed complications) across postoperative days for patients with (**a**) complicated appendicitis (41 events) and (**b**) simple appendicitis (10 events). Events shown in red were missed by the model (i.e., neither the day prior to the event, nor two days prior to the event were flagged). Events shown in gray did not have data available for model training because they occurred on either the first postoperative day or occurred immediately after another postoperative events (i.e., there were no days *prior* to the abnormal event available for early detection, so these events were not represented in algorithm training and testing). All other events were successfully detected by the model up to 1-2 days prior to the event.

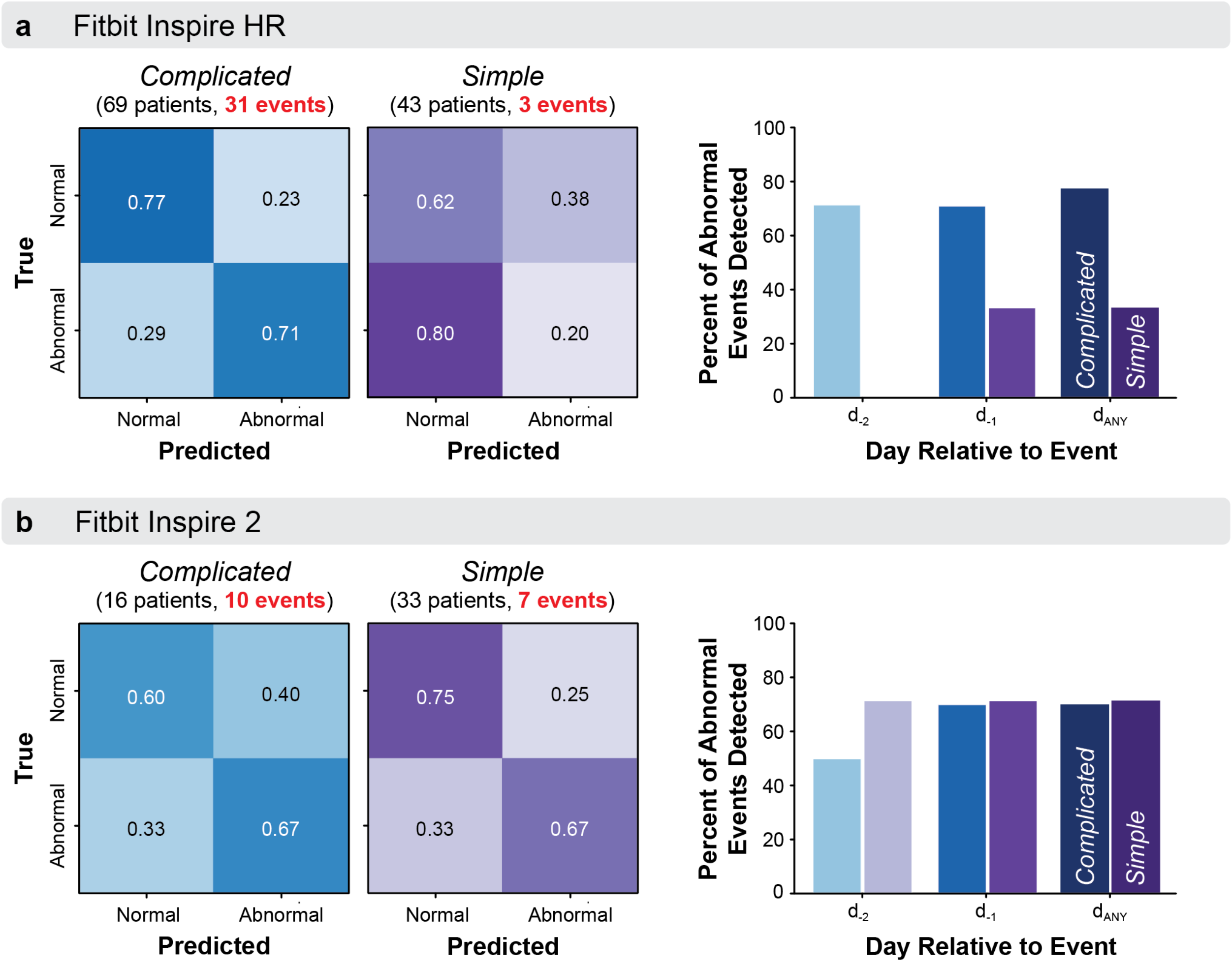

Supplementary Figure 3: Sensitivity analysis of Fitbit device type. Model performance for participants undergoing appendectomy for complicated and simple appendicitis using data collected from (**a**) only the Inspire HR (112 patients) and (**b**) only the Inspire 2 (49 patients). For complicated appendicitis, there are substantially fewer postoperative events available for model training and testing with the Inspire 2 compared to the Inspire HR. Conversely, for simple appendicitis, there are fewer events for the Inspire HR (only three) compared to the Inspire 2.

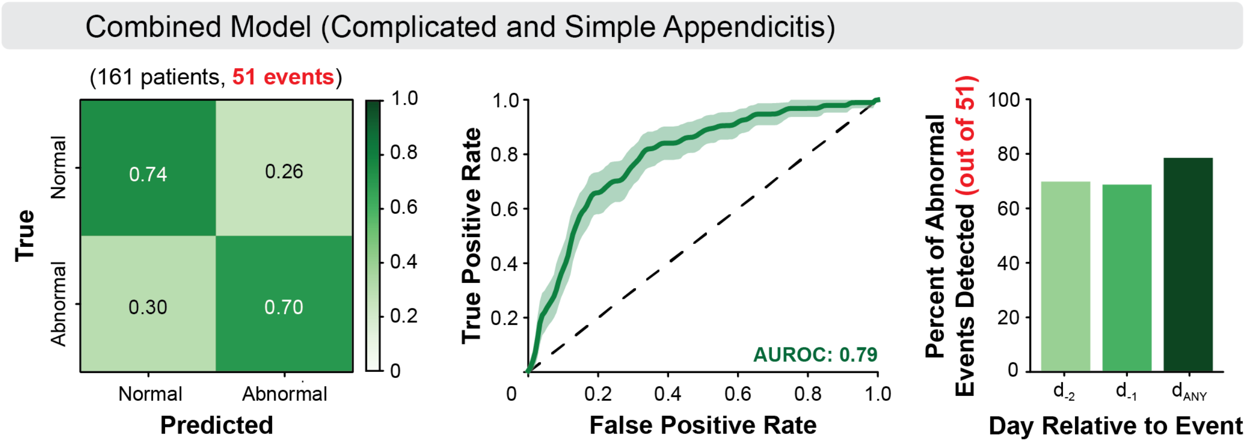

Supplementary Figure 4: Sensitivity analysis of combined appendicitis groups. Confusion matrix, receiver operating characteristic, and percent of postoperative events (either confirmed complications or abnormal symptoms) detected for all participants, including patients with complicated appendicitis and patients with simple appendicitis.
