# Supplementary material for: Applying machine learning to consumer wearable data for the early detection of complications after pediatric appendectomy
Source: NPJ Digit Med. 2023 Aug 16;6:148. doi: 10.1038/s41746-023-00890-z (PMC10432429; doi:10.1038/s41746-023-00890-z)
Supplement: Supplementary file 2 — Reporting Summary [file 41746_2023_890_MOESM2_ESM.pdf]

Corresponding author(s): Fizan Abdullah, MD, PhD

Last updated by author(s): Jul 3, 2023

Reporting Summary

Nature Portfolio wishes to improve the reproducibility of the work that we publish. This form provides structure for consistency and transparency in reporting. For further information on Nature Portfolio policies, see our Editorial Policies and the Editorial Policy Checklist.

Statistics

For all statistical analyses, confirm that the following items are present in the figure legend, table legend, main text, or Methods section.

n/a	Confirmed	
☐	☒	The exact sample size (*n*) for each experimental group/condition, given as a discrete number and unit of measurement
☐	☒	A statement on whether measurements were taken from distinct samples or whether the same sample was measured repeatedly
☐	☒	The statistical test(s) used AND whether they are one- or two-sided<br>*Only common tests should be described solely by name; describe more complex techniques in the Methods section.*
☒	☐	A description of all covariates tested
☒	☐	A description of any assumptions or corrections, such as tests of normality and adjustment for multiple comparisons
☐	☒	A full description of the statistical parameters including central tendency (e.g. means) or other basic estimates (e.g. regression coefficient) AND variation (e.g. standard deviation) or associated estimates of uncertainty (e.g. confidence intervals)
☒	☐	For null hypothesis testing, the test statistic (e.g. *F*, *t*, *r*) with confidence intervals, effect sizes, degrees of freedom and *P* value noted<br>*Give P values as exact values whenever suitable.*
☒	☐	For Bayesian analysis, information on the choice of priors and Markov chain Monte Carlo settings
☒	☐	For hierarchical and complex designs, identification of the appropriate level for tests and full reporting of outcomes
☒	☐	Estimates of effect sizes (e.g. Cohen's *d*, Pearson's *r*), indicating how they were calculated

Our web collection on statistics for biologists contains articles on many of the points above.

Software and code

Policy information about availability of computer code

Data collection	Proprietary software used by the Fitbit Inspire HR and Fitbit Inspire 2 wearable devices automatically computed activity, HR, and sleep metrics which could be obtained from the Fitbit devices. Proprietary software by Fitabase, using a Fitbit Partner API, was used to collect and store this data for subsequent analysis.
Data analysis	Machine learning analysis was conducted using Python 3.8.5 with packages Scikit-learn 1.1.1; imbalanced-learn 0.9.1.

For manuscripts utilizing custom algorithms or software that are central to the research but not yet described in published literature, software must be made available to editors and reviewers. We strongly encourage code deposition in a community repository (e.g. GitHub). See the Nature Portfolio guidelines for submitting code & software for further information.

Data

Policy information about availability of data

All manuscripts must include a data availability statement. This statement should provide the following information, where applicable:

- Accession codes, unique identifiers, or web links for publicly available datasets
- A description of any restrictions on data availability
- For clinical datasets or third party data, please ensure that the statement adheres to our policy

The clinical data used in this study belongs to the Ann and Robert H. Lurie Children’s Hospital of Chicago, and restrictions apply to the availability of these data.

Qualified researchers affiliated with the Ann and Robert H. Lurie Children's Hospital of Chicago may apply for access to these data through the Ann and Robert H. Lurie Children's Hospital of Chicago institutional review board.

Research involving human participants, their data, or biological material

Policy information about studies with human participants or human data. See also policy information about sex, gender (identity/presentation), and sexual orientation and race, ethnicity and racism.

Reporting on sex and gender	Sex of our cohort was reported in order to describe our study patient population. Results of this study are applicable to both males and females.
Reporting on race, ethnicity, or other socially relevant groupings	The Race/Ethnicity of our cohort was considered as a feature in our machine learning algorithm. In addition, it was reported in order to describe the demographics of our study's population. This was collected from the patient's electronic medical record which is patient/parent reported. Patients were classified as White, non-Hispanic, Hispanic, African American or Other. Other included Asian, American Indian/Alaskan Native, Other, Unknown, Not Documented and Declined and was included to represent a grouping of our least represented patients.
Population characteristics	The following demographics are described in Table 1<br>Patient age at time of surgery<br>Patient sex (male/female)<br>Patient race/ethnicity
Recruitment	Patients and their caregivers were recruited shortly after surgery, and written informed consent, using IRB-approved forms was obtained from a parent or legal guardian for all children <18 years old. In addition, oral assent was obtained from patients 7-11 years old and written assent from patients 12 years and older. Patients were given the FItbit after study completion as renumeration for participation. . Recruitment was limited to working days of the week, and there was a period of pause in recruitment. However, given the emergent nature of appendectomy, we anticipate that appendicitis patients who had their operation earlier in the study or those who had their operation on a weekend would have demographic and complication profiles similar to the study population. Furthermore, near real-time Fitbit monitoring would require access to a cell phone with a data plan for online synchronization with a clinical database. Though all caregivers approached in the study had a cell phone with a data plan, this requirement may be a barrier to participation for some patients and caregivers.
Ethics oversight	Ann & Robert H Lurie Children's Hospital of Chicago

Note that full information on the approval of the study protocol must also be provided in the manuscript.

Field-specific reporting

Please select the one below that is the best fit for your research. If you are not sure, read the appropriate sections before making your selection.

☒ Life sciences ☐ Behavioural & social sciences ☐ Ecological, evolutionary & environmental sciences

For a reference copy of the document with all sections, see nature.com/documents/nr-reporting-summary-flat.pdf

Life sciences study design

All studies must disclose on these points even when the disclosure is negative.

Sample size	161
Data exclusions	Non-ambulatory children and children with preexisting mobility limitations or postoperative activity limitations, children with comorbidities that could alter the postoperative course, and children with COVID-19 were excluded. One patient was excluded from analysis due to only having one hour of Fitbit data.
Replication	A leave-one-subject-out cross validation was used to estimate the mean and standard deviation of model outcome measures to assess their consistency across participants. Additionally, multiple training strategies and machine learning models were tested to replicate the analysis about predictive value of Fitbit data. Although performance of the individual models varied (as is standard in machine learning, since the algorithms learn differently from the data and many of them include a level of stochasticity), the overall findings that Fitbit data improved model predictions, and that the effect was more pronounced for complicated appendicitis compared to simple appendicitis, held for all analyses. Multiple random seeds were tested for stochastic models during testing to ensure replication. After this, the random seed was set to ensure reproducibility of the final models presented in this study.
Randomization	n/a
Blinding	n/a

Reporting for specific materials, systems and methods

We require information from authors about some types of materials, experimental systems and methods used in many studies. Here, indicate whether each material, system or method listed is relevant to your study. If you are not sure if a list item applies to your research, read the appropriate section before selecting a response.

Materials & experimental systems

n/a	Involved in the study
☒	☐ Antibodies
☒	☐ Eukaryotic cell lines
☒	☐ Palaeontology and archaeology
☒	☐ Animals and other organisms
☒	☐ Clinical data
☒	☐ Dual use research of concern
☒	☐ Plants

Methods

n/a	Involved in the study
☒	☐ ChIP-seq
☒	☐ Flow cytometry
☒	☐ MRI-based neuroimaging
